# Public health round-up

**DOI:** 10.2471/BLT.26.010726

**Published:** 2026-07-01

**Preventing tobacco youth use**

Around 40 million children aged 13 to 15 years use tobacco products worldwide, and the use of e‑cigarettes and nicotine pouches among young people continues to rise. The World Health Organization (WHO) is urging governments to act to prevent a new generation from becoming addicted. It warns that tobacco and nicotine companies are designing products to be more appealing, especially for adolescents. Nicotine is highly addictive and particularly harmful to developing brains. WHO urges governments to curb youth use by banning flavoured products, restricting advertising and sponsorship, enforcing smoke‑ and vape‑free indoor spaces, and strengthening enforcement measures.

**Figure:**
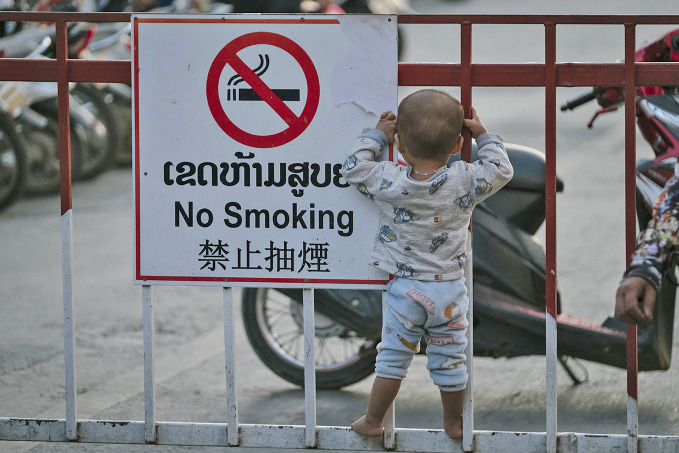
WHO/FCTC/Vlad Sokhin

## Ebola response plan

The Africa Centres for Disease Control and Prevention (Africa CDC) and the World Health Organization (WHO) have launched a joint continental plan to address the Ebola outbreak caused by the Bundibugyo virus. The six-month initiative (June to November 2026) seeks 518 million United States dollars to strengthen preparedness and response across African countries through a unified approach. It focuses on coordination, surveillance, laboratory testing, infection control, clinical care, community engagement, logistics and sustaining essential health services.

The plan complements national strategies in the Democratic Republic of the Congo and Uganda, while emphasizing protection of vulnerable populations, cross-border collaboration and rapid response to emerging cases. It also aims to reinforce health systems in the absence of licensed vaccines or therapeutics for this Ebola strain.

 “Ebola moves fast. Africa must move faster. This joint plan gives the continent a clear path to act with speed and unity: to save lives, support the affected countries and protect neighbouring communities. With Member States, WHO and partners, Africa CDC is turning commitment into action and resources into response for the communities at risk,” said Jean Kaseya, Director-General of Africa CDC.

Drawing on lessons learned from previous Ebola outbreaks and other public health emergencies, the plan also provides a pathway to broadly strengthen Africa’s capacity to prevent, detect and respond to future health threats while protecting lives and livelihoods. 

https://bit.ly/4oA9stM

## Guidelines on filovirus disease

WHO has released its first comprehensive guidelines for the clinical management of filovirus diseases, including Ebola and Marburg, amid an ongoing Bundibugyo Ebola outbreak in the Democratic Republic of the Congo. The guidelines present 16 evidence-based recommendations, emphasizing early supportive care to improve survival and health outcomes, especially in the absence of licensed vaccines or treatments for some filovirus strains.

Filovirus diseases are severe and often fatal, with case fatality rates ranging from 25% to 90%. Since 1967, Africa has recorded 72 outbreaks of Ebola and Marburg, causing significant social, economic and psychological impacts. The new guidance draws on lessons from past outbreaks and the latest scientific evidence to improve clinical care and response readiness.

“These new guidelines are a perfect example of how WHO leverages science to better protect and care for people during outbreaks and health emergencies,” said WHO Director-General Tedros Adhanom Ghebreyesus.

Developed through global expert consultations and based on the most up-to-date scientific evidence and clinical knowledge, the guidelines translate lessons learned from recent Ebola and Marburg disease outbreaks into practical recommendations for improved patient care. Overall, the guidelines aim to support frontline health workers, standardize care and strengthen health system preparedness.

https://bit.ly/4voEjMG

## Step-by-Step tool

In collaboration with the Pan American Health Organization (PAHO) and Lebanon’s National Mental Health Programme, WHO presented its digital intervention Step-by-Step at the World Bank Group’s Fragility Forum in Washington, DC, United States of America. The event gathered over 1500 participants from governments, international organizations and civil society, and highlighted innovative solutions for fragile and crisis-affected settings. 

Step-by-Step is a free, WHO-developed digital self-help tool designed to help people manage depression and anxiety with light support from trained non-specialists such as community health workers. The program uses a picture-based narrative and includes five modules, complemented by brief weekly check-ins.

Clinical trials in Lebanon involving nearly 1250 Lebanese and Syrian participants demonstrated significant reductions in depression, anxiety and stress, along with improved daily functioning. Nearly half of Lebanese users and over one third of Syrian users experienced a reduction in depressive symptoms of more than 50%, compared to only about 14% among non-users.

Now implemented nationwide in Lebanon and Thailand, and adapted in India, Step-by-Step offers an accessible, cost-effective mental health solution globally.

https://bit.ly/4a9vHB1

## Pharmaceutical decarbonization 

Global stakeholders convened by WHO have called for harmonized standards, stronger collaboration, and practical regulatory pathways to support the transition to lower‑carbon pharmaceuticals without compromising access, quality or safety. The WHO-led forum brought together over 100 participants from more than 30 organizations, including regulators, health agencies, industry, academia and global partners, to examine how regulation can accelerate pharmaceutical decarbonization. Participants explored how regulatory systems can help reduce the environmental footprint of medicines while maintaining the core mandate of ensuring safe, effective and quality-assured products.

“Climate change and health is a strategic priority for WHO,” said Rogério Gaspar, director of WHO’s Department of Regulation and Prequalification. “The objective of this initiative is to clarify where regulation currently constrains or slows down decarbonization; identify opportunities for regulatory leadership and flexibility; and build consensus across regulators, industry and global health actors on practical pathways that preserve quality, safety and access.”

A key outcome of the forum was the recognition that regulators play a critical role in enabling greener pharmaceutical practices. Participants highlighted that existing regulatory tools can already support sustainability while emphasizing the need to balance environmental goals with equitable access to essential medicines, particularly in low- and middle-income countries.

https://bit.ly/3SByH2Z

## Blood safety and availability

The recently published *Global status report on blood safety and availability 2025* from WHO shows steady global progress in blood safety and availability, alongside persistent inequalities and systemic challenges. The report draws on data from 168 countries, covering 97% of the world’s population, and provides the most comprehensive global assessment of blood systems to date. 

Between 2013 and 2023, blood collections rose by nearly 19%, reaching about 120 million donations annually, with over 85% coming from voluntary unpaid donors. Despite this progress, access remains uneven, especially in low-income countries where patients facing childbirth complications, severe anaemia, trauma or chronic conditions, often lack timely transfusions. Major disparities persist, with high-income countries collecting 36% of global donations despite having only 15% of the population. Weak governance, limited financing and gaps in regulation further hinder equitable access, underscoring the need for stronger national blood systems and sustained investment. 

“Governments must continue investing in strong, sustainable national blood systems and supporting the voluntary unpaid blood donors whose generosity saves millions of lives every year," said Tedros Adhanom Ghebreyesus, WHO Director-General 

https://bit.ly/4ewfhE9

## Andes hantavirus research initiative

A globally coordinated research initiative has been launched following the Andes hantavirus (ANDV) outbreak linked to the *MV Hondius* cruise ship, demonstrating how rapidly international research systems can be activated during health emergencies. Known as the Natural History Study of Andes Virus Infection (NAVIS), the study involves institutions from 21 countries and aims to better understand ANDV transmission, immune responses, disease severity and viral behaviour through standardized, longitudinal follow-up of exposed individuals.

The initiative will use a harmonized protocol developed by Hospital Germans Trias i Pujol in Spain, for immediate deployment after an emergency scientific consultation, which engaged over 1600 experts from more than 130 countries to identify urgent research priorities. 

By applying consistent methods across countries, NAVIS seeks to generate comparable data to inform the development of diagnostics, treatments and vaccines. The project uses the International Severe Acute Respiratory and Emerging Infection Consortium (ISARIC) framework for rapid, standardized data collection.

NAVIS highlights the importance of preparedness, emphasizing the need for pre-established research systems that can be activated immediately and for strong global collaboration.

“Scientific evidence generation during outbreaks must become operational, coordinated and immediately deployable. Future outbreak responses should begin by activating research systems that already exist rather than trying to build them during crises,” said Sylvie Briand, WHO’s Chief Scientist. 

https://bit.ly/44pdVGl

**Cover photo**

Emergency supplies being mobilized from the WHO Emergency Preparedness and Response Hub warehouse in Nairobi, Kenya in response to the ongoing Ebola outbreak caused by Bundibugyo virus in the Democratic Republic of the Congo.

**Figure:**
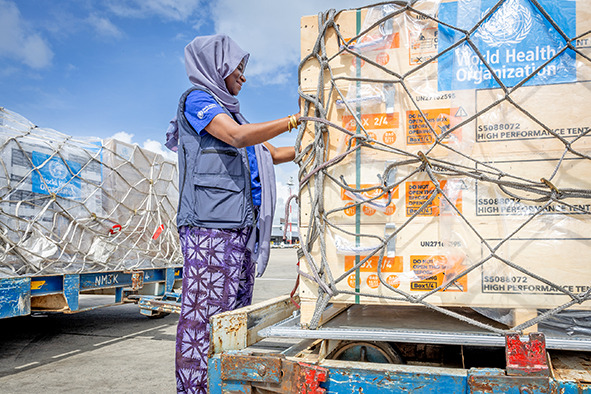
WHO

**Looking ahead**

6–9 July. WHO Product Development for Vaccines Advisory Committee Meeting. Geneva, Switzerland. https://bit.ly/4vTN5SA

20–21 July. United Nations High-Level Meeting on Improving Global Road Safety. New York, United States of America https://bit.ly/4vfOJhn

